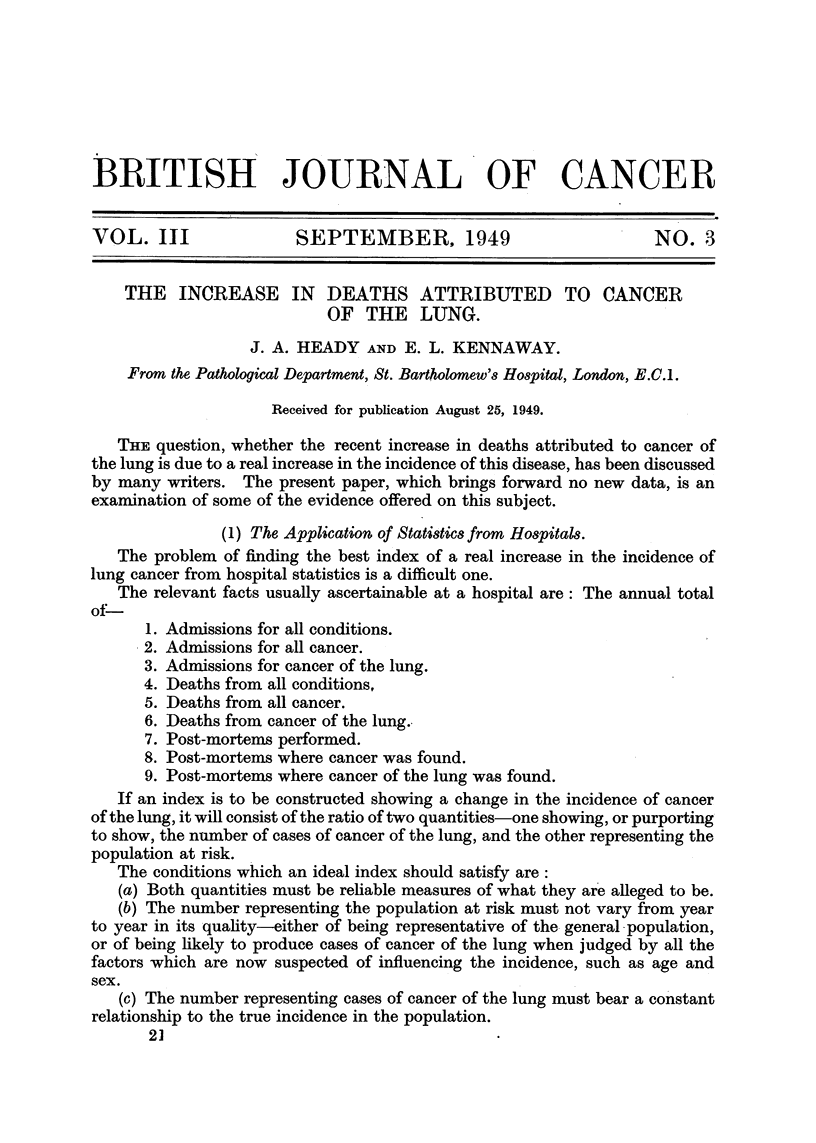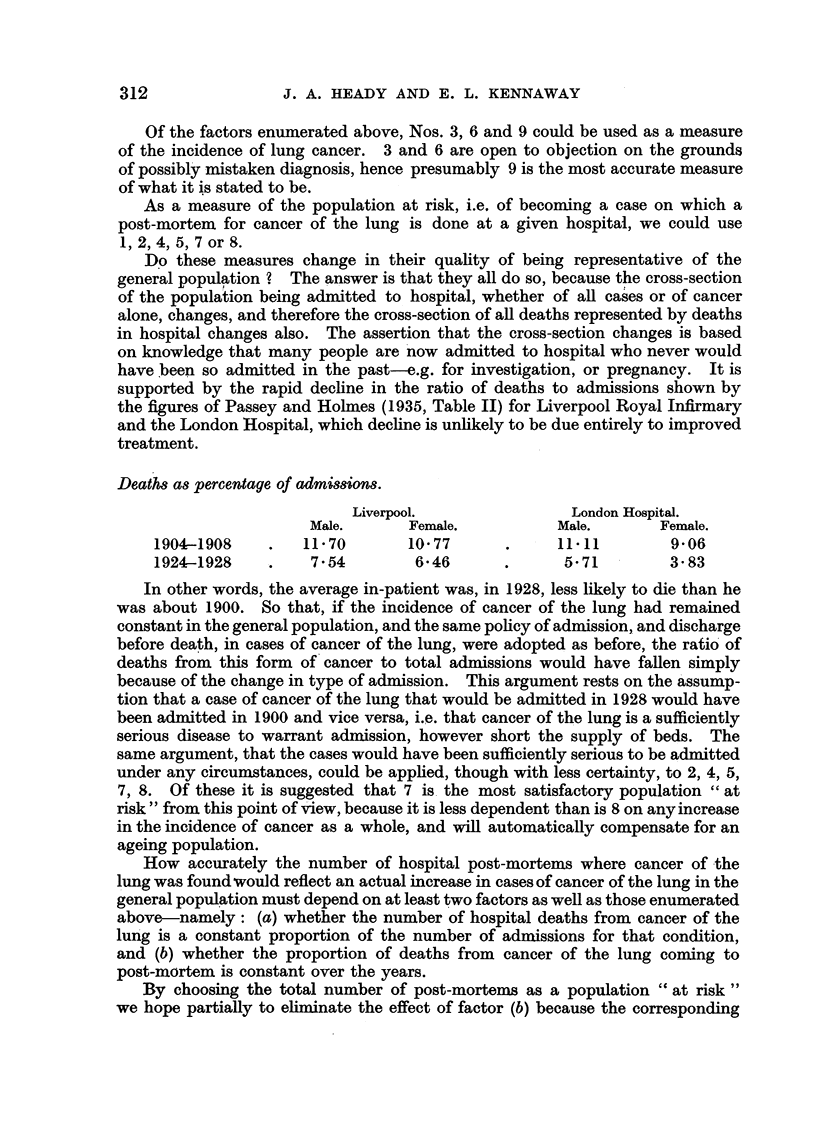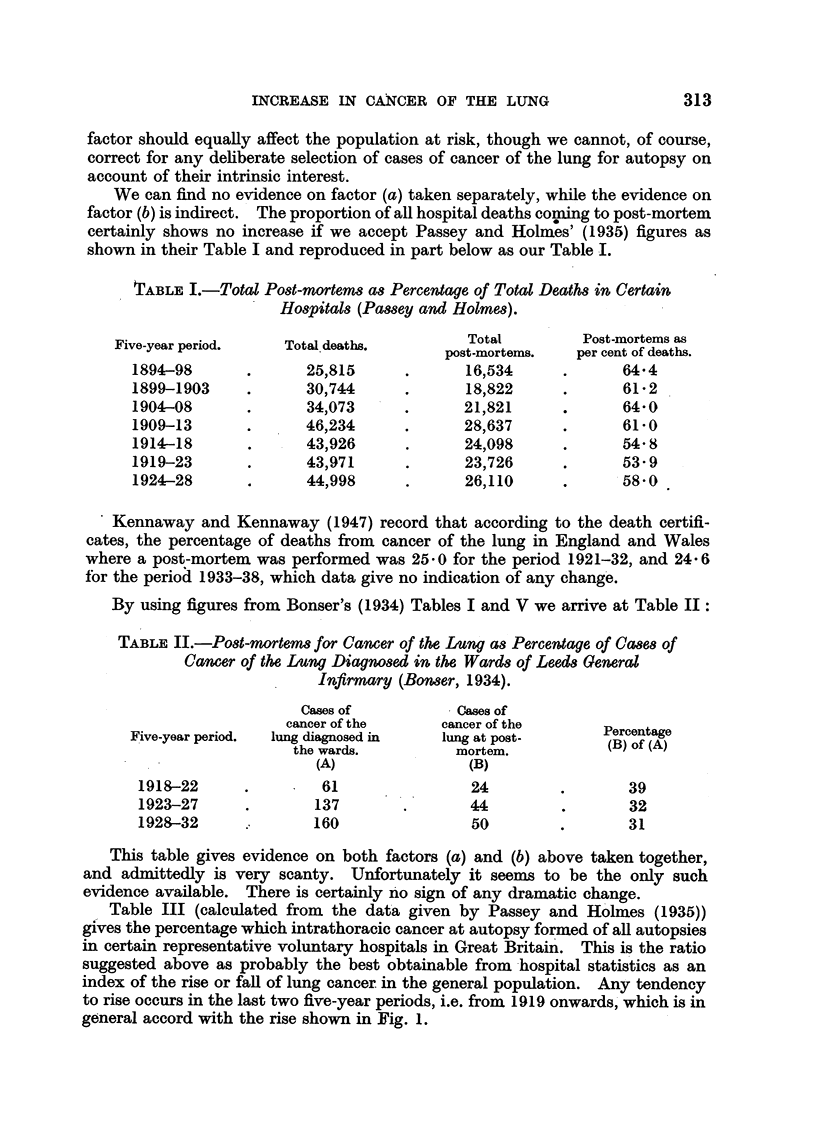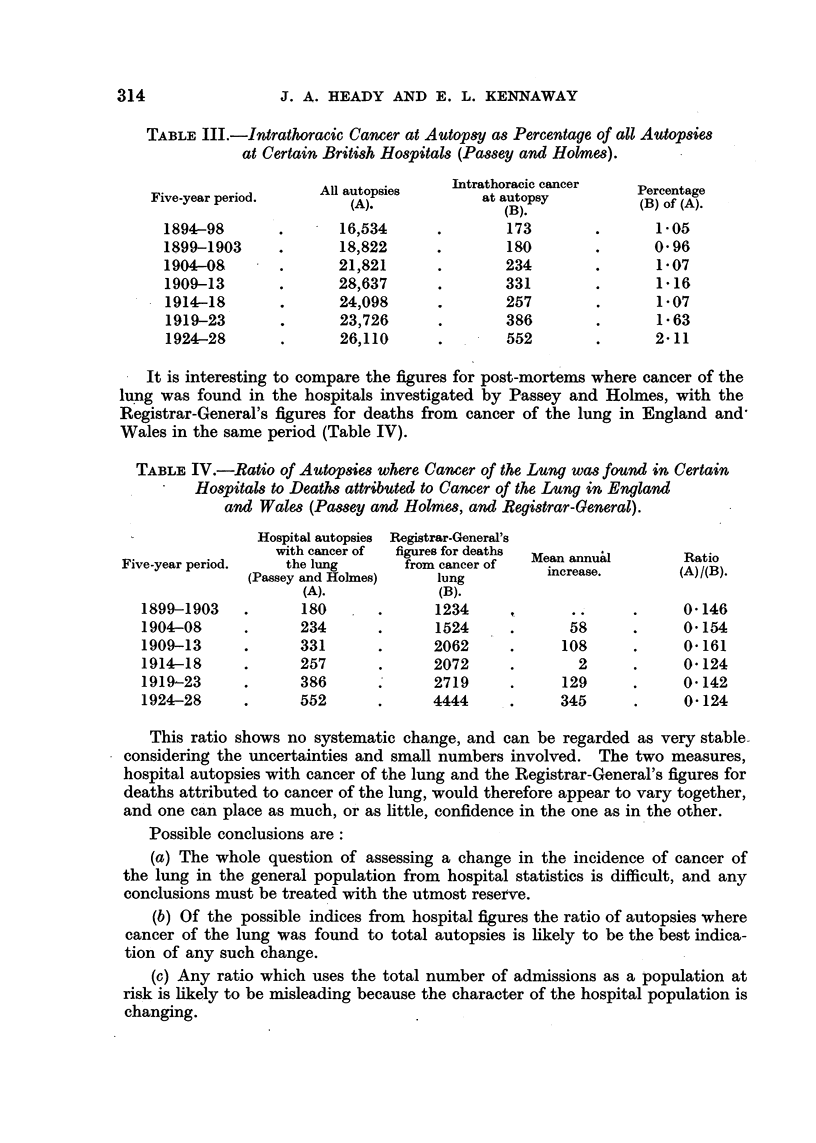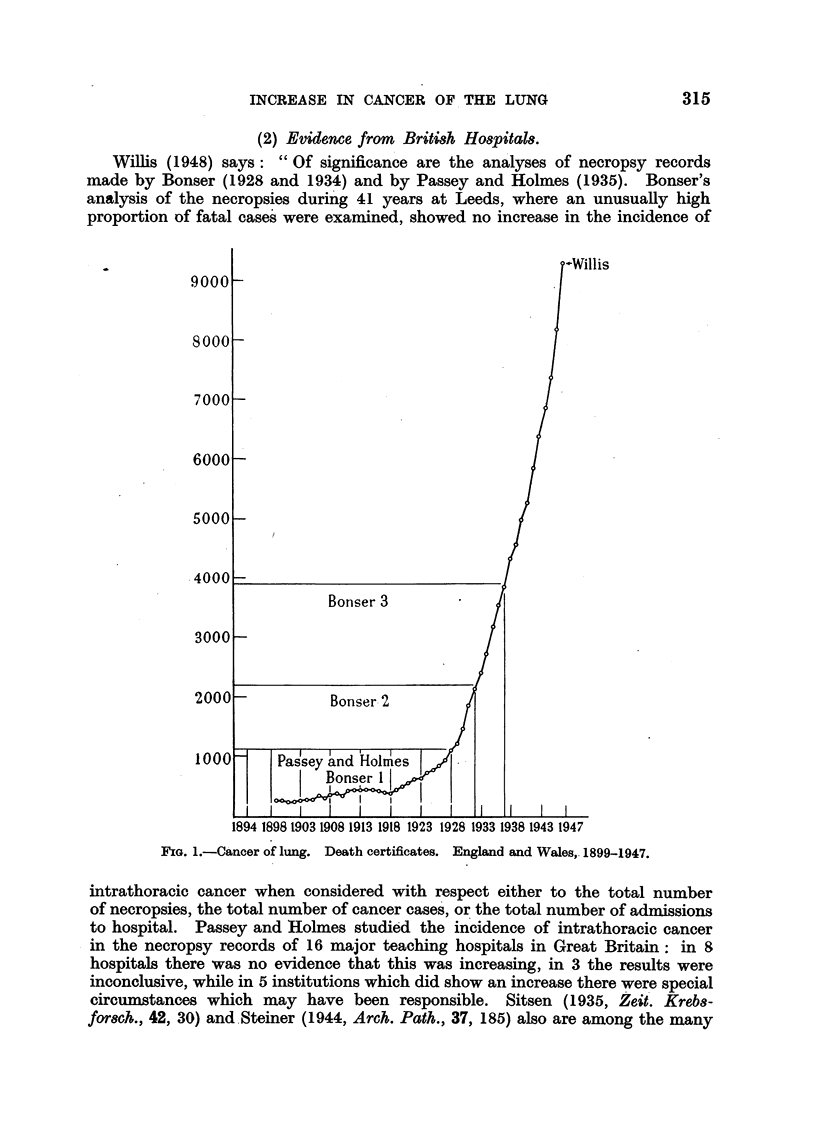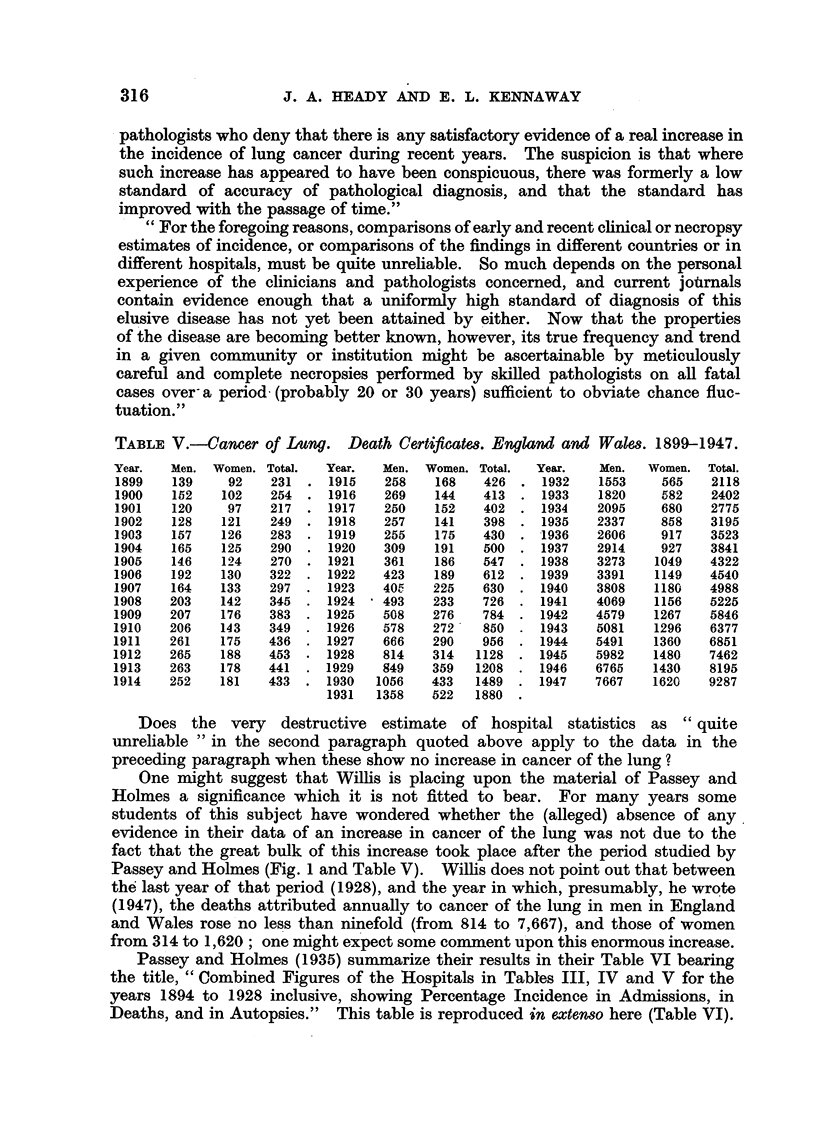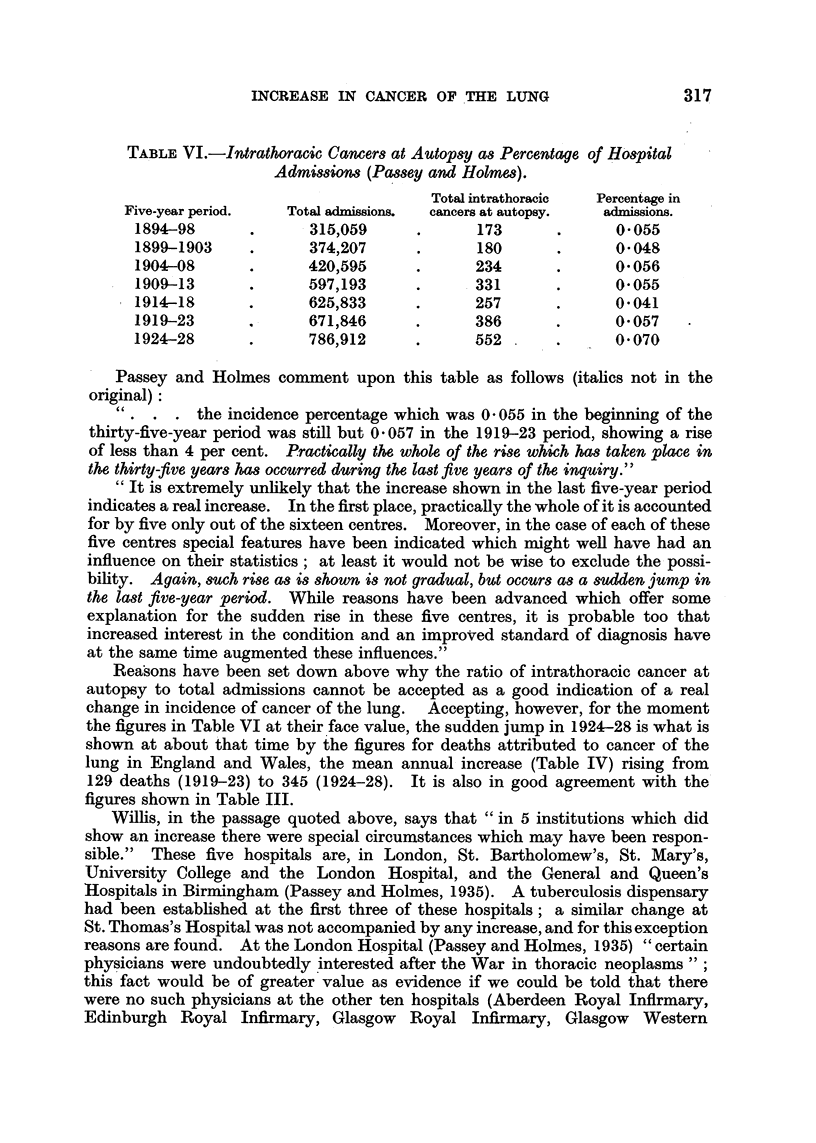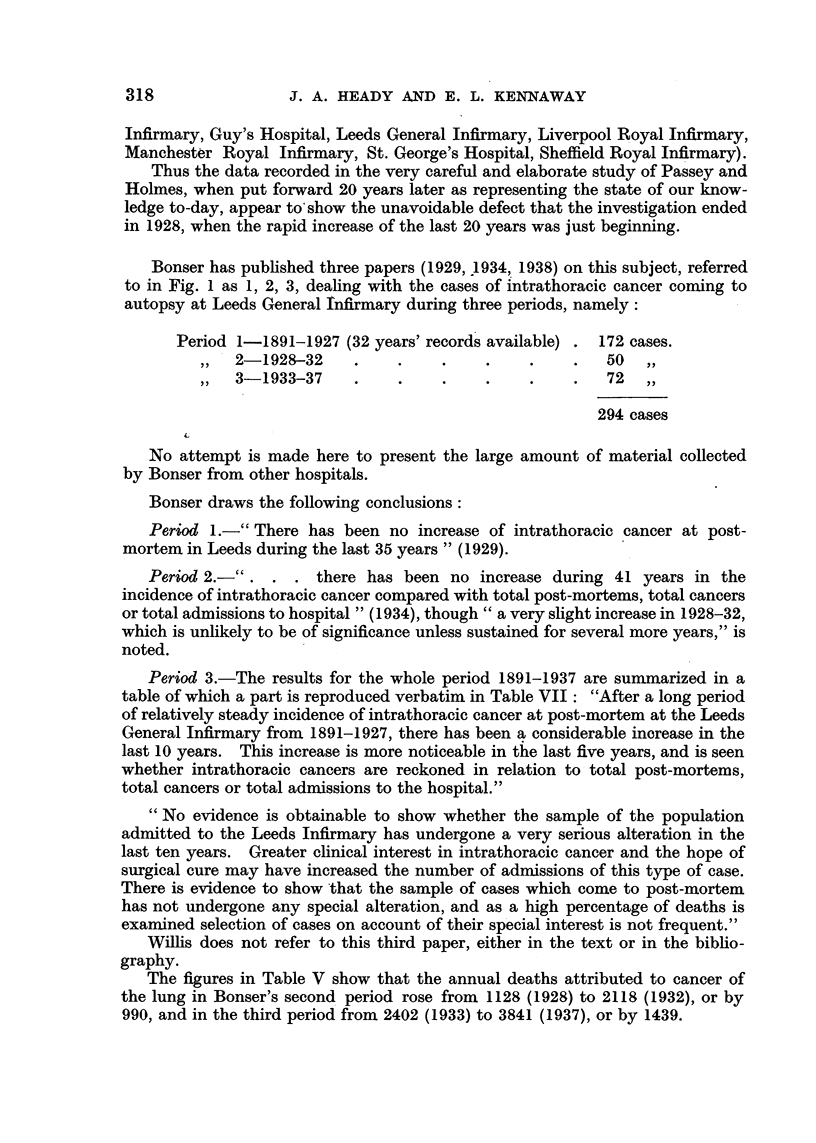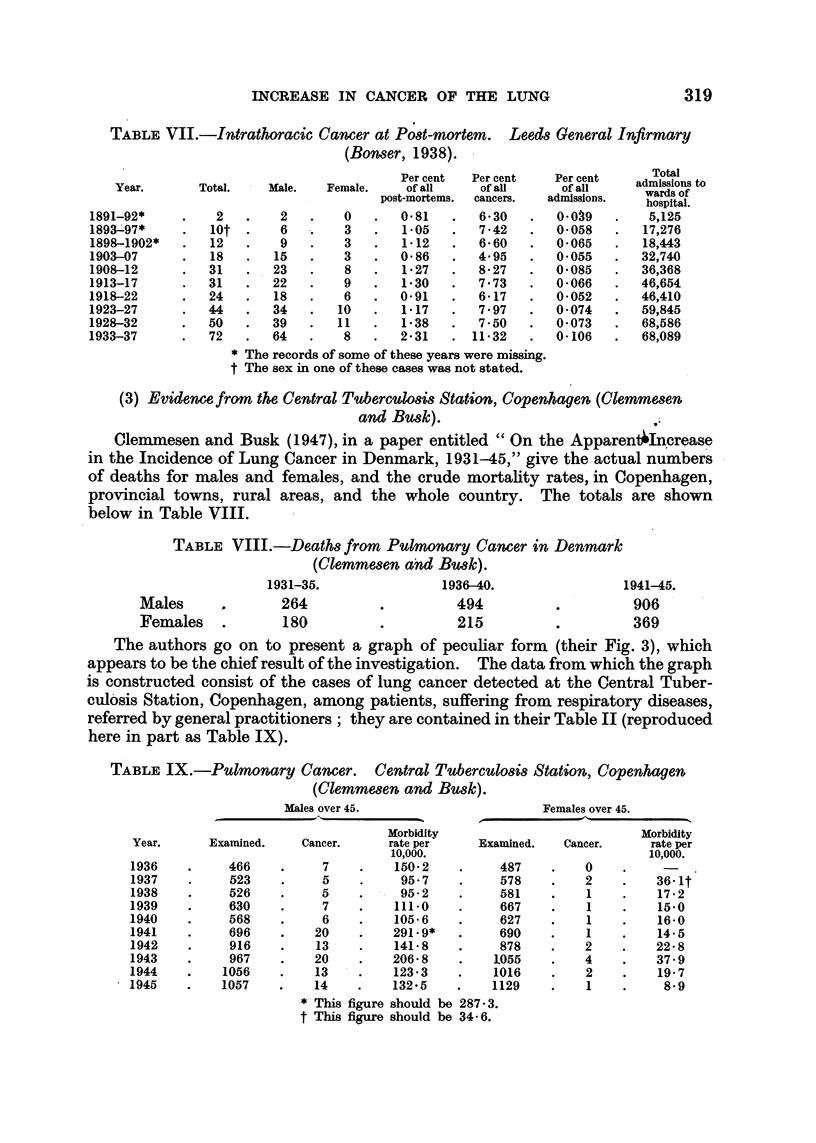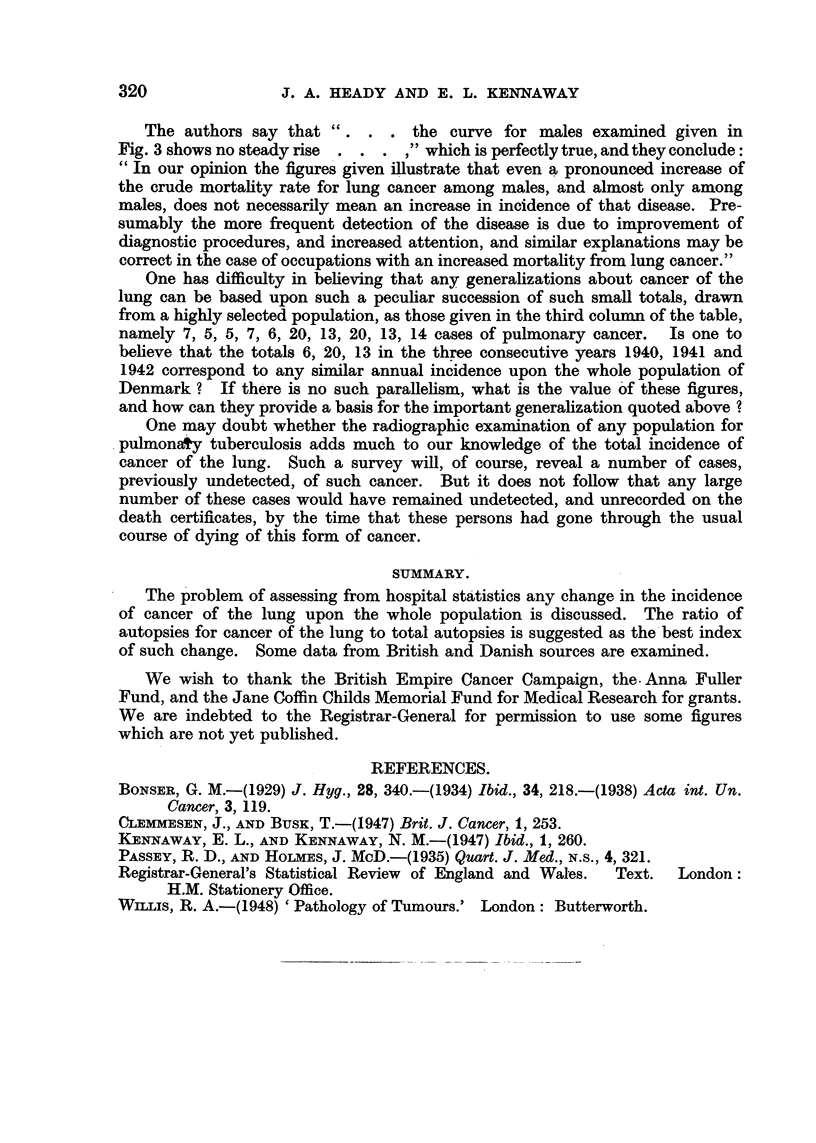# The Increase in Deaths Attributed to Cancer of the Lung

**DOI:** 10.1038/bjc.1949.37

**Published:** 1949-09

**Authors:** J. A. Heady, E. L. Kennaway


BRITISH JOURNAL OF CANCER

VOL. III SEPTEMBER, 1949 NO. 3

### THE INCREASE IN DEATHS ATTRIBUTED TO CANCER OF THE LUNG.

J. A. HEADY AND E. L. KENNAWAY.

*From the Pathological Department, St. Bartholomew's Hospital, London, E.C.*1.

Received for publication August 25, 1949.

THE question, whether the recent increase in deaths attributed to cancer of the lung is due to a real increase in the incidence of this disease, has been discussed by many writers. The present paper, which brings forward no new data, is an examination of some of the evidence offered on this subject.

### (1) *The Application of Statistics from Hospitals.*

The problem of finding the best index of a real increase in the incidence of lung cancer from hospital statistics is a difficult one.

The relevant facts usually ascertainable at a hospital are : The annual total of—

1. Admissions for all conditions.
2. Admissions for all cancer.
3. Admissions for cancer of the lung.
4. Deaths from all conditions.
5. Deaths from all cancer.
6. Deaths from cancer of the lung.
7. Post-mortems performed.
8. Post-mortems where cancer was found.
9. Post-mortems where cancer of the lung was found.

If an index is to be constructed showing a change in the incidence of cancer of the lung, it will consist of the ratio of two quantities—one showing, or purporting to show, the number of cases of cancer of the lung, and the other representing the population at risk.

The conditions which an ideal index should satisfy are :

(*a*) Both quantities must be reliable measures of what they are alleged to be.

(*b*) The number representing the population at risk must not vary from year to year in its quality—either of being representative of the general population, or of being likely to produce cases of cancer of the lung when judged by all the factors which are now suspected of influencing the incidence, such as age and sex.

(*c*) The number representing cases of cancer of the lung must bear a constant relationship to the true incidence in the population.

Of the factors enumerated above, Nos. 3, 6 and 9 could be used as a measure of the incidence of lung cancer. 3 and 6 are open to objection on the grounds of possibly mistaken diagnosis, hence presumably 9 is the most accurate measure of what it is stated to be.

As a measure of the population at risk, i.e. of becoming a case on which a post-mortem for cancer of the lung is done at a given hospital, we could use 1, 2, 4, 5, 7 or 8.

Do these measures change in their quality of being representative of the general population ? The answer is that they all do so, because the cross-section of the population being admitted to hospital, whether of all cases or of cancer alone, changes, and therefore the cross-section of all deaths represented by deaths in hospital changes also. The assertion that the cross-section changes is based on knowledge that many people are now admitted to hospital who never would have been so admitted in the past—e.g. for investigation, or pregnancy. It is supported by the rapid decline in the ratio of deaths to admissions shown by the figures of Passey and Holmes (1935, Table II) for Liverpool Royal Infirmary and the London Hospital, which decline is unlikely to be due entirely to improved treatment.

*Deaths as percentage of admissions.*

| | Liverpool. | | London Hospital. | |
|---|---|---|---|---|
| | Male. | Female. | Male. | Female. |
| 1904–1908 . | 11·70 | 10·77 . | 11·11 | 9·06 |
| 1924–1928 . | 7·54 | 6·46 . | 5·71 | 3·83 |

In other words, the average in-patient was, in 1928, less likely to die than he was about 1900. So that, if the incidence of cancer of the lung had remained constant in the general population, and the same policy of admission, and discharge before death, in cases of cancer of the lung, were adopted as before, the ratio of deaths from this form of cancer to total admissions would have fallen simply because of the change in type of admission. This argument rests on the assumption that a case of cancer of the lung that would be admitted in 1928 would have been admitted in 1900 and vice versa, i.e. that cancer of the lung is a sufficiently serious disease to warrant admission, however short the supply of beds. The same argument, that the cases would have been sufficiently serious to be admitted under any circumstances, could be applied, though with less certainty, to 2, 4, 5, 7, 8. Of these it is suggested that 7 is the most satisfactory population " at risk " from this point of view, because it is less dependent than is 8 on any increase in the incidence of cancer as a whole, and will automatically compensate for an ageing population.

How accurately the number of hospital post-mortems where cancer of the lung was found would reflect an actual increase in cases of cancer of the lung in the general population must depend on at least two factors as well as those enumerated above—namely : (*a*) whether the number of hospital deaths from cancer of the lung is a constant proportion of the number of admissions for that condition, and (*b*) whether the proportion of deaths from cancer of the lung coming to post-mortem is constant over the years.

By choosing the total number of post-mortems as a population " at risk " we hope partially to eliminate the effect of factor (*b*) because the corresponding factor should equally affect the population at risk, though we cannot, of course, correct for any deliberate selection of cases of cancer of the lung for autopsy on account of their intrinsic interest.

We can find no evidence on factor (*a*) taken separately, while the evidence on factor (*b*) is indirect. The proportion of all hospital deaths coming to post-mortem certainly shows no increase if we accept Passey and Holmes' (1935) figures as shown in their Table I and reproduced in part below as our Table I.

TABLE I.—*Total Post-mortems as Percentage of Total Deaths in Certain Hospitals* (*Passey and Holmes*).

| Five-year period. | Total deaths. | Total post-mortems. | Post-mortems as per cent of deaths. |
|---|---|---|---|
| 1894–98 | 25,815 | 16,534 | 64·4 |
| 1899–1903 | 30,744 | 18,822 | 61·2 |
| 1904–08 | 34,073 | 21,821 | 64·0 |
| 1909–13 | 46,234 | 28,637 | 61·0 |
| 1914–18 | 43,926 | 24,098 | 54·8 |
| 1919–23 | 43,971 | 23,726 | 53·9 |
| 1924–28 | 44,998 | 26,110 | 58·0 |

Kennaway and Kennaway (1947) record that according to the death certificates, the percentage of deaths from cancer of the lung in England and Wales where a post-mortem was performed was 25·0 for the period 1921–32, and 24·6 for the period 1933–38, which data give no indication of any change.

By using figures from Bonser's (1934) Tables I and V we arrive at Table II:

TABLE II.—*Post-mortems for Cancer of the Lung as Percentage of Cases of Cancer of the Lung Diagnosed in the Wards of Leeds General Infirmary* (*Bonser*, 1934).

| Five-year period. | Cases of cancer of the lung diagnosed in the wards. (A) | Cases of cancer of the lung at post-mortem. (B) | Percentage (B) of (A) |
|---|---|---|---|
| 1918–22 | 61 | 24 | 39 |
| 1923–27 | 137 | 44 | 32 |
| 1928–32 | 160 | 50 | 31 |

This table gives evidence on both factors (*a*) and (*b*) above taken together, and admittedly is very scanty. Unfortunately it seems to be the only such evidence available. There is certainly no sign of any dramatic change.

Table III (calculated from the data given by Passey and Holmes (1935)) gives the percentage which intrathoracic cancer at autopsy formed of all autopsies in certain representative voluntary hospitals in Great Britain. This is the ratio suggested above as probably the best obtainable from hospital statistics as an index of the rise or fall of lung cancer in the general population. Any tendency to rise occurs in the last two five-year periods, i.e. from 1919 onwards, which is in general accord with the rise shown in Fig. 1.

TABLE III.—*Intrathoracic Cancer at Autopsy as Percentage of all Autopsies at Certain British Hospitals (Passey and Holmes).*

| Five-year period. | All autopsies (A). | Intrathoracic cancer at autopsy (B). | Percentage (B) of (A). |
|---|---|---|---|
| 1894–98 | 16,534 | 173 | 1·05 |
| 1899–1903 | 18,822 | 180 | 0·96 |
| 1904–08 | 21,821 | 234 | 1·07 |
| 1909–13 | 28,637 | 331 | 1·16 |
| 1914–18 | 24,098 | 257 | 1·07 |
| 1919–23 | 23,726 | 386 | 1·63 |
| 1924–28 | 26,110 | 552 | 2·11 |

It is interesting to compare the figures for post-mortems where cancer of the lung was found in the hospitals investigated by Passey and Holmes, with the Registrar-General's figures for deaths from cancer of the lung in England and Wales in the same period (Table IV).

TABLE IV.—*Ratio of Autopsies where Cancer of the Lung was found in Certain Hospitals to Deaths attributed to Cancer of the Lung in England and Wales (Passey and Holmes, and Registrar-General).*

| Five-year period. | Hospital autopsies with cancer of the lung (Passey and Holmes) (A). | Registrar-General's figures for deaths from cancer of lung (B). | Mean annual increase. | Ratio (A)/(B). |
|---|---|---|---|---|
| 1899–1903 | 180 | 1234 | .. | 0·146 |
| 1904–08 | 234 | 1524 | 58 | 0·154 |
| 1909–13 | 331 | 2062 | 108 | 0·161 |
| 1914–18 | 257 | 2072 | 2 | 0·124 |
| 1919–23 | 386 | 2719 | 129 | 0·142 |
| 1924–28 | 552 | 4444 | 345 | 0·124 |

This ratio shows no systematic change, and can be regarded as very stable considering the uncertainties and small numbers involved. The two measures, hospital autopsies with cancer of the lung and the Registrar-General's figures for deaths attributed to cancer of the lung, would therefore appear to vary together, and one can place as much, or as little, confidence in the one as in the other.

Possible conclusions are :

(*a*) The whole question of assessing a change in the incidence of cancer of the lung in the general population from hospital statistics is difficult, and any conclusions must be treated with the utmost reserve.

(*b*) Of the possible indices from hospital figures the ratio of autopsies where cancer of the lung was found to total autopsies is likely to be the best indication of any such change.

(*c*) Any ratio which uses the total number of admissions as a population at risk is likely to be misleading because the character of the hospital population is changing.

(2) *Evidence from British Hospitals.*

Willis (1948) says : " Of significance are the analyses of necropsy records made by Bonser (1928 and 1934) and by Passey and Holmes (1935). Bonser's analysis of the necropsies during 41 years at Leeds, where an unusually high proportion of fatal cases were examined, showed no increase in the incidence of

FIG. 1.—Cancer of lung. Death certificates. England and Wales, 1899–1947.

intrathoracic cancer when considered with respect either to the total number of necropsies, the total number of cancer cases, or the total number of admissions to hospital. Passey and Holmes studied the incidence of intrathoracic cancer in the necropsy records of 16 major teaching hospitals in Great Britain : in 8 hospitals there was no evidence that this was increasing, in 3 the results were inconclusive, while in 5 institutions which did show an increase there were special circumstances which may have been responsible. Sitsen (1935, *Zeit. Krebsforsch.*, **42**, 30) and Steiner (1944, *Arch. Path.*, **37**, 185) also are among the many pathologists who deny that there is any satisfactory evidence of a real increase in the incidence of lung cancer during recent years. The suspicion is that where such increase has appeared to have been conspicuous, there was formerly a low standard of accuracy of pathological diagnosis, and that the standard has improved with the passage of time."

" For the foregoing reasons, comparisons of early and recent clinical or necropsy estimates of incidence, or comparisons of the findings in different countries or in different hospitals, must be quite unreliable. So much depends on the personal experience of the clinicians and pathologists concerned, and current journals contain evidence enough that a uniformly high standard of diagnosis of this elusive disease has not yet been attained by either. Now that the properties of the disease are becoming better known, however, its true frequency and trend in a given community or institution might be ascertainable by meticulously careful and complete necropsies performed by skilled pathologists on all fatal cases over a period (probably 20 or 30 years) sufficient to obviate chance fluctuation."

TABLE V.—*Cancer of Lung. Death Certificates. England and Wales.* 1899–1947.

| Year. | Men. | Women. | Total. | Year. | Men. | Women. | Total. | Year. | Men. | Women. | Total. |
|---|---|---|---|---|---|---|---|---|---|---|---|
| 1899 | 139 | 92 | 231 | 1915 | 258 | 168 | 426 | 1932 | 1553 | 565 | 2118 |
| 1900 | 152 | 102 | 254 | 1916 | 269 | 144 | 413 | 1933 | 1820 | 582 | 2402 |
| 1901 | 120 | 97 | 217 | 1917 | 250 | 152 | 402 | 1934 | 2095 | 680 | 2775 |
| 1902 | 128 | 121 | 249 | 1918 | 257 | 141 | 398 | 1935 | 2337 | 858 | 3195 |
| 1903 | 157 | 126 | 283 | 1919 | 255 | 175 | 430 | 1936 | 2606 | 917 | 3523 |
| 1904 | 165 | 125 | 290 | 1920 | 309 | 191 | 500 | 1937 | 2914 | 927 | 3841 |
| 1905 | 146 | 124 | 270 | 1921 | 361 | 186 | 547 | 1938 | 3273 | 1049 | 4322 |
| 1906 | 192 | 130 | 322 | 1922 | 423 | 189 | 612 | 1939 | 3391 | 1149 | 4540 |
| 1907 | 164 | 133 | 297 | 1923 | 405 | 225 | 630 | 1940 | 3808 | 1180 | 4988 |
| 1908 | 203 | 142 | 345 | 1924 | 493 | 233 | 726 | 1941 | 4069 | 1156 | 5225 |
| 1909 | 207 | 176 | 383 | 1925 | 508 | 276 | 784 | 1942 | 4579 | 1267 | 5846 |
| 1910 | 206 | 143 | 349 | 1926 | 578 | 272 | 850 | 1943 | 5081 | 1296 | 6377 |
| 1911 | 261 | 175 | 436 | 1927 | 666 | 290 | 956 | 1944 | 5491 | 1360 | 6851 |
| 1912 | 265 | 188 | 453 | 1928 | 814 | 314 | 1128 | 1945 | 5982 | 1480 | 7462 |
| 1913 | 263 | 178 | 441 | 1929 | 849 | 359 | 1208 | 1946 | 6765 | 1430 | 8195 |
| 1914 | 252 | 181 | 433 | 1930 | 1056 | 433 | 1489 | 1947 | 7667 | 1620 | 9287 |
| | | | | 1931 | 1358 | 522 | 1880 | | | | |

Does the very destructive estimate of hospital statistics as " quite unreliable " in the second paragraph quoted above apply to the data in the preceding paragraph when these show no increase in cancer of the lung ?

One might suggest that Willis is placing upon the material of Passey and Holmes a significance which it is not fitted to bear. For many years some students of this subject have wondered whether the (alleged) absence of any evidence in their data of an increase in cancer of the lung was not due to the fact that the great bulk of this increase took place after the period studied by Passey and Holmes (Fig. 1 and Table V). Willis does not point out that between the last year of that period (1928), and the year in which, presumably, he wrote (1947), the deaths attributed annually to cancer of the lung in men in England and Wales rose no less than ninefold (from 814 to 7,667), and those of women from 314 to 1,620 ; one might expect some comment upon this enormous increase.

Passey and Holmes (1935) summarize their results in their Table VI bearing the title, " Combined Figures of the Hospitals in Tables III, IV and V for the years 1894 to 1928 inclusive, showing Percentage Incidence in Admissions, in Deaths, and in Autopsies." This table is reproduced *in extenso* here (Table VI).

TABLE VI.—*Intrathoracic Cancers at Autopsy as Percentage of Hospital Admissions (Passey and Holmes).*

| Five-year period. | Total admissions. | Total intrathoracic cancers at autopsy. | Percentage in admissions. |
|---|---|---|---|
| 1894–98 | 315,059 | 173 | 0·055 |
| 1899–1903 | 374,207 | 180 | 0·048 |
| 1904–08 | 420,595 | 234 | 0·056 |
| 1909–13 | 597,193 | 331 | 0·055 |
| 1914–18 | 625,833 | 257 | 0·041 |
| 1919–23 | 671,846 | 386 | 0·057 |
| 1924–28 | 786,912 | 552 | 0·070 |

Passey and Holmes comment upon this table as follows (italics not in the original):

" . . . the incidence percentage which was 0·055 in the beginning of the thirty-five-year period was still but 0·057 in the 1919–23 period, showing a rise of less than 4 per cent. *Practically the whole of the rise which has taken place in the thirty-five years has occurred during the last five years of the inquiry.*"

" It is extremely unlikely that the increase shown in the last five-year period indicates a real increase. In the first place, practically the whole of it is accounted for by five only out of the sixteen centres. Moreover, in the case of each of these five centres special features have been indicated which might well have had an influence on their statistics ; at least it would not be wise to exclude the possibility. *Again, such rise as is shown is not gradual, but occurs as a sudden jump in the last five-year period.* While reasons have been advanced which offer some explanation for the sudden rise in these five centres, it is probable too that increased interest in the condition and an improved standard of diagnosis have at the same time augmented these influences."

Reasons have been set down above why the ratio of intrathoracic cancer at autopsy to total admissions cannot be accepted as a good indication of a real change in incidence of cancer of the lung. Accepting, however, for the moment the figures in Table VI at their face value, the sudden jump in 1924–28 is what is shown at about that time by the figures for deaths attributed to cancer of the lung in England and Wales, the mean annual increase (Table IV) rising from 129 deaths (1919–23) to 345 (1924–28). It is also in good agreement with the figures shown in Table III.

Willis, in the passage quoted above, says that " in 5 institutions which did show an increase there were special circumstances which may have been responsible." These five hospitals are, in London, St. Bartholomew's, St. Mary's, University College and the London Hospital, and the General and Queen's Hospitals in Birmingham (Passey and Holmes, 1935). A tuberculosis dispensary had been established at the first three of these hospitals ; a similar change at St. Thomas's Hospital was not accompanied by any increase, and for this exception reasons are found. At the London Hospital (Passey and Holmes, 1935) "certain physicians were undoubtedly interested after the War in thoracic neoplasms" ; this fact would be of greater value as evidence if we could be told that there were no such physicians at the other ten hospitals (Aberdeen Royal Infirmary, Edinburgh Royal Infirmary, Glasgow Royal Infirmary, Glasgow Western

Infirmary, Guy's Hospital, Leeds General Infirmary, Liverpool Royal Infirmary, Manchester Royal Infirmary, St. George's Hospital, Sheffield Royal Infirmary).

Thus the data recorded in the very careful and elaborate study of Passey and Holmes, when put forward 20 years later as representing the state of our knowledge to-day, appear to show the unavoidable defect that the investigation ended in 1928, when the rapid increase of the last 20 years was just beginning.

Bonser has published three papers (1929, 1934, 1938) on this subject, referred to in Fig. 1 as 1, 2, 3, dealing with the cases of intrathoracic cancer coming to autopsy at Leeds General Infirmary during three periods, namely :

| | |
|---|---|
| Period 1—1891–1927 (32 years' records available) . | 172 cases. |
| ,, 2—1928–32 . . . . . . . | 50 ,, |
| ,, 3—1933–37 . . . . . . . | 72 ,, |
| | 294 cases |

No attempt is made here to present the large amount of material collected by Bonser from other hospitals.

Bonser draws the following conclusions :

*Period* 1.—" There has been no increase of intrathoracic cancer at post-mortem in Leeds during the last 35 years " (1929).

*Period* 2.—" . . . there has been no increase during 41 years in the incidence of intrathoracic cancer compared with total post-mortems, total cancers or total admissions to hospital " (1934), though " a very slight increase in 1928–32, which is unlikely to be of significance unless sustained for several more years," is noted.

*Period* 3.—The results for the whole period 1891–1937 are summarized in a table of which a part is reproduced verbatim in Table VII : "After a long period of relatively steady incidence of intrathoracic cancer at post-mortem at the Leeds General Infirmary from 1891–1927, there has been a considerable increase in the last 10 years. This increase is more noticeable in the last five years, and is seen whether intrathoracic cancers are reckoned in relation to total post-mortems, total cancers or total admissions to the hospital."

" No evidence is obtainable to show whether the sample of the population admitted to the Leeds Infirmary has undergone a very serious alteration in the last ten years. Greater clinical interest in intrathoracic cancer and the hope of surgical cure may have increased the number of admissions of this type of case. There is evidence to show that the sample of cases which come to post-mortem has not undergone any special alteration, and as a high percentage of deaths is examined selection of cases on account of their special interest is not frequent."

Willis does not refer to this third paper, either in the text or in the bibliography.

The figures in Table V show that the annual deaths attributed to cancer of the lung in Bonser's second period rose from 1128 (1928) to 2118 (1932), or by 990, and in the third period from 2402 (1933) to 3841 (1937), or by 1439.

TABLE VII.—*Intrathoracic Cancer at Post-mortem. Leeds General Infirmary (Bonser, 1938).*

| Year. | Total. | Male. | Female. | Per cent of all post-mortems. | Per cent of all cancers. | Per cent of all admissions. | Total admissions to wards of hospital. |
|---|---|---|---|---|---|---|---|
| 1891–92* | 2 | 2 | 0 | 0·81 | 6·30 | 0·039 | 5,125 |
| 1893–97* | 10† | 6 | 3 | 1·05 | 7·42 | 0·058 | 17,276 |
| 1898–1902* | 12 | 9 | 3 | 1·12 | 6·60 | 0·065 | 18,443 |
| 1903–07 | 18 | 15 | 3 | 0·86 | 4·95 | 0·055 | 32,740 |
| 1908–12 | 31 | 23 | 8 | 1·27 | 8·27 | 0·085 | 36,368 |
| 1913–17 | 31 | 22 | 9 | 1·30 | 7·73 | 0·066 | 46,654 |
| 1918–22 | 24 | 18 | 6 | 0·91 | 6·17 | 0·052 | 46,410 |
| 1923–27 | 44 | 34 | 10 | 1·17 | 7·97 | 0·074 | 59,845 |
| 1928–32 | 50 | 39 | 11 | 1·38 | 7·50 | 0·073 | 68,586 |
| 1933–37 | 72 | 64 | 8 | 2·31 | 11·32 | 0·106 | 68,089 |

* The records of some of these years were missing.
† The sex in one of these cases was not stated.

(3) *Evidence from the Central Tuberculosis Station, Copenhagen (Clemmesen and Busk).*

Clemmesen and Busk (1947), in a paper entitled "On the Apparent Increase in the Incidence of Lung Cancer in Denmark, 1931–45," give the actual numbers of deaths for males and females, and the crude mortality rates, in Copenhagen, provincial towns, rural areas, and the whole country. The totals are shown below in Table VIII.

TABLE VIII.—*Deaths from Pulmonary Cancer in Denmark (Clemmesen and Busk).*

| | 1931–35. | 1936–40. | 1941–45. |
|---|---|---|---|
| Males | 264 | 494 | 906 |
| Females | 180 | 215 | 369 |

The authors go on to present a graph of peculiar form (their Fig. 3), which appears to be the chief result of the investigation. The data from which the graph is constructed consist of the cases of lung cancer detected at the Central Tuberculosis Station, Copenhagen, among patients, suffering from respiratory diseases, referred by general practitioners ; they are contained in their Table II (reproduced here in part as Table IX).

TABLE IX.—*Pulmonary Cancer. Central Tuberculosis Station, Copenhagen (Clemmesen and Busk).*

| | Males over 45. | | | Females over 45. | | |
|---|---|---|---|---|---|---|
| Year. | Examined. | Cancer. | Morbidity rate per 10,000. | Examined. | Cancer. | Morbidity rate per 10,000. |
| 1936 | 466 | 7 | 150·2 | 487 | 0 | — |
| 1937 | 523 | 5 | 95·7 | 578 | 2 | 36·1† |
| 1938 | 526 | 5 | 95·2 | 581 | 1 | 17·2 |
| 1939 | 630 | 7 | 111·0 | 667 | 1 | 15·0 |
| 1940 | 568 | 6 | 105·6 | 627 | 1 | 16·0 |
| 1941 | 696 | 20 | 291·9* | 690 | 1 | 14·5 |
| 1942 | 916 | 13 | 141·8 | 878 | 2 | 22·8 |
| 1943 | 967 | 20 | 206·8 | 1055 | 4 | 37·9 |
| 1944 | 1056 | 13 | 123·3 | 1016 | 2 | 19·7 |
| 1945 | 1057 | 14 | 132·5 | 1129 | 1 | 8·9 |

* This figure should be 287·3.
† This figure should be 34·6.

The authors say that " . . . the curve for males examined given in Fig. 3 shows no steady rise . . . ," which is perfectly true, and they conclude: " In our opinion the figures given illustrate that even a pronounced increase of the crude mortality rate for lung cancer among males, and almost only among males, does not necessarily mean an increase in incidence of that disease. Presumably the more frequent detection of the disease is due to improvement of diagnostic procedures, and increased attention, and similar explanations may be correct in the case of occupations with an increased mortality from lung cancer."

One has difficulty in believing that any generalizations about cancer of the lung can be based upon such a peculiar succession of such small totals, drawn from a highly selected population, as those given in the third column of the table, namely 7, 5, 5, 7, 6, 20, 13, 20, 13, 14 cases of pulmonary cancer. Is one to believe that the totals 6, 20, 13 in the three consecutive years 1940, 1941 and 1942 correspond to any similar annual incidence upon the whole population of Denmark ? If there is no such parallelism, what is the value of these figures, and how can they provide a basis for the important generalization quoted above ?

One may doubt whether the radiographic examination of any population for pulmonary tuberculosis adds much to our knowledge of the total incidence of cancer of the lung. Such a survey will, of course, reveal a number of cases, previously undetected, of such cancer. But it does not follow that any large number of these cases would have remained undetected, and unrecorded on the death certificates, by the time that these persons had gone through the usual course of dying of this form of cancer.

### SUMMARY.

The problem of assessing from hospital statistics any change in the incidence of cancer of the lung upon the whole population is discussed. The ratio of autopsies for cancer of the lung to total autopsies is suggested as the best index of such change. Some data from British and Danish sources are examined.

We wish to thank the British Empire Cancer Campaign, the Anna Fuller Fund, and the Jane Coffin Childs Memorial Fund for Medical Research for grants. We are indebted to the Registrar-General for permission to use some figures which are not yet published.

### REFERENCES.

Bonser, G. M.—(1929) *J. Hyg.*, **28**, 340.—(1934) *Ibid.*, **34**, 218.—(1938) *Acta int. Un. Cancer*, **3**, 119.

Clemmesen, J., and Busk, T.—(1947) *Brit. J. Cancer*, **1**, 253.

Kennaway, E. L., and Kennaway, N. M.—(1947) *Ibid.*, **1**, 260.

Passey, R. D., and Holmes, J. McD.—(1935) *Quart. J. Med.*, n.s., **4**, 321.

Registrar-General's Statistical Review of England and Wales. Text. London: H.M. Stationery Office.

Willis, R. A.—(1948) 'Pathology of Tumours.' London: Butterworth.